# Acceptability and effectiveness of the “Education in Action—ABALL1” intervention program in primary school-aged children

**DOI:** 10.3389/fpsyg.2023.1163489

**Published:** 2023-07-20

**Authors:** Isabel S. Silva, Filipa Cunha-Saraiva, Sandra Silvestre

**Affiliations:** ^1^Grupo Aprender em Festa, Gouveia, Portugal; ^2^RECI-Research Unit in Education and Community Intervention, Instituto Piaget-ISEIT/Viseu, Viseu, Portugal; ^3^Centre for Functional Ecology-Science for People & the Planet (CFE), TERRA Associate Laboratory, Department of Life Sciences, University of Coimbra (UC), Coimbra, Portugal

**Keywords:** acceptability, learning, play, education, literacy, numeracy, socioemotional skills, children

## Abstract

**Background:**

Research has consistently shown the benefits of developing intervention programs in educational settings, enhancing the learning process and socioemotional skills. There is a growing investment in creating and supporting a healthy school environment, prioritizing learning through play. This study aimed to assess the acceptability and effectiveness of an innovative intervention approach—“Education in Action—ABALL1”—focused on promoting literacy and numeracy skills and socioemotional competencies of second-grade children.

**Methods:**

A total of 113 children aged between 7 and 9 participated in the study and were allocated into two groups: intervention (*N* = 69) and control (*N* = 44). The intervention consisted of 24 educational games aligned with the mathematics and Portuguese curricula, applied for 3 months, twice a week; two self-report instruments were used to measure aptitudes for school learning and socioemotional skills, considering two assessment moments: before and after the program implementation. In addition, a focus group involving a subsample of children and teachers who followed the intervention in different school cohorts was carried out.

**Results:**

Our results suggested a positive effect of the program concerning children's academic skills, showing a significant improvement in terms of the pre–post-intervention scores in the intervention group (Cohen's *d* = 0.95). Moreover, the qualitative findings also indicate the high acceptability of the program among children and head teachers, who reported a positive effect on the acquisition and consolidation of reading, writing, and arithmetic skills and on the promotion of teamwork, empathy, autonomy, and self-reflection.

**Conclusion:**

Overall, the “Education in Action—ABALL1” program provides a promising intervention based on learning through play directly impacting second-grade children's academic, emotional, and interpersonal skills. Further studies are required to understand the transdisciplinary capacity of this intervention approach and its effectiveness at different school levels and curricula.

## 1. Introduction

The first years of a child's life play a pivotal role in their development (Gleitman et al., 2011). The theory of cognitive development, proposed by Piaget, is divided into four stages (Piaget and Inhelder, 1969; Ginsburg and Opper, 1988; Singer and Revenson, 1997). As a child enters the stage of concrete operative thinking (7 to 11 years old), processes such as logical reasoning begin to emerge. Academic skills such as reading and writing open the child's perspective to their surroundings. During this stage, essential experiences for cognitive development contemplate active learning, such as overt motor behavior (e.g., Singer and Revenson, 1997). Learning concepts are paramount for a child's school performance. However, children need to be able to relate the acquired content to its applicability in contexts outside the school environment (Martins et al., 2017; Zosh et al., 2017). Therefore, the ability to self-coordinate cognitive, emotional, and behavioral aspects in diverse contexts is essential to overcome personal and social challenges at the family, school, and community levels (Greenberg et al., 2017).

School programs or strategies that promote the acquisition and development of emotional intelligence allow future adults to become competent in their social, emotional, and academic skills, and make them more resilient in their problem-solving ability (Mahoney et al., 2021). These aptitudes can be retained through playful experiences, and it is up to the teacher/facilitator/parent to address children's natural curiosity and enhance their creativity and critical thinking.

Learning through play is a survival mechanism that allows the acquisition of important competencies for the transition from the juvenile stage to adulthood (Spinka et al., 2001; Bruce, 2011). Previous studies have shown the beneficial outcomes of play activities on playful behavior in children, such as reduced cortisol levels (Potasz et al., 2013; Carro et al., 2023) or improved social behavior and cognitive skills (Henniger, 1995; Farmer-Dougan and Kaszuba, 1999) Lived experiences during childhood exercise the cognitive muscle, which is plastic and adaptive, allowing for proper neurological and motor development (Brown and Vaughan, 2009; Bruce, 2011). Skills including language and/or logical-mathematical abilities are extensively worked on and reinforced during moments of *free play*.

Children's naturally triggered play activities (*free play*) contribute to their enjoyment but are also catalyzing events for collaborative and communicative skills (Zosh et al., 2017; OECD, 2021). Moreover, they promote cognitive competency, emotional regulation, and the development of a social repertoire, which in turn will enhance children's school performance (Zosh et al., 2017). The act of playing encourages children's active learning and autonomy (Daniels and Shumow, 2003). Therefore, when elementary school children can choose the game to play and understand and assimilate its characteristics, they are more motivated to play and learn from it (Cordova and Lepper, 1996).

Educational games (here defined as a physical or mental contest played according to specific rules, with the goal of amusing or rewarding the participant; Noemí and Máximo, 2014), digital or physical, effectively engage and motivate students to learn, unlike a strictly conventional educational environment (Rosas et al., 2003; Wrzesien and Raya, 2010). School environments that acknowledge the student as an active participant in the learning process (i.e., student-focused school environments) exhibit an increased motivation to learn (Stipek et al., 1995), increased problem-solving, and assertive communication skills (Stipek et al., 1998).

Subsequently, educational games are a relevant pedagogical tool, promoting several aspects related to understanding instructions, achievement of goals, sensory stimulation, and motivation for learning (Garris et al., 2002). The physical component of educational games, such as aerobic exercises (running and jumping), increases blood flow stimulating complex cognitive processes (Best, 2010). This increased cognitive ability enables a correct attitude and posture at school (Riggs et al., 2004) and improves the child's emotional regulation (Blair and Diamond, 2008).

The applicability of educational games is numerous across subjects and contexts and may be associated with literacy and numeracy skill enhancement (Morgan and Fuchs, 2007; Ke and Abras, 2013). Several studies show that using educational games in preschool (Fisher et al., 2013) and primary school (Ronimus et al., 2019) improves the application of theoretical knowledge. In the school context, a space is provided where children feel involved in a positive and inclusive environment, with diverse social interactions, where they can learn and be creative through play (Arends, 2011; OECD, 2021).

Developed in Norway by Andersen and Sandnes (2014), the educational game, ABALL1, is mainly implemented in dynamics with young people in situations of social vulnerability, and of reception and integration in refugee camps, to promote academic, social, and physical competencies across all ability levels.

Approved by the Portuguese Government in 2017 (Martins et al., 2017), the twenty-first-century children's profile is a reference document for the organization of the entire educational system, contributing to the convergence and articulation of decisions inherent to the various dimensions of curriculum development. Therefore, it provides a school and learning environment in which the students of this global generation build and consolidate a scientific and artistic culture with a humanistic base. To this end, they learn and acquire values and skills that enable them to make free and informed decisions and ethical choices and to have a capacity for active, conscious, and responsible civic participation.

Recognizing the educational and pedagogical potential of the game ABALL1 for the Portuguese school context, the program “Education in Action—ABALL1” emerges from the need to provide an educational tool to respond more effectively to the characteristics and profiles of twenty-first-century children (Martins et al., 2017). The “Education in Action—ABALL1” program, despite having games from the original Norwegian tool, is pioneering, in the sense that the program includes not only the translation and cultural validation of those games to the Portuguese school context but also has new games aligned with the syllabus of the Portuguese educational system.

Hence, the aims of this study are as follows: (i) to assess the acceptability and effectiveness of a new intervention program “Educação em Ação—ABALL1” and its ability to promote literacy, numeracy, and socioemotional skills in second-grade children and (ii) to explore the relationship between learning and socioemotional competencies.

## 2. Materials and methods

### 2.1. Translation and cultural adaptation of the program

The “Education in Action—ABALL1” program was developed based on the educational tool—ABALL1 comprising 13 different game plans.

Following the international guidelines, the translation and cultural adaptation process of this educational tool was conducted in six phases (Hambleton, 2015): (1) translation of ABALL1 to Portuguese; (2) content adaptation; (3) independent evaluation of the cultural and semantic adaptability by experts; (4) appraisal by the target population (e.g., second-grade children); (5) systematization of recommendations and improvement of contents; and (6) implementation and assessment of acceptability and effectiveness of the program.

More specifically, the translation process involved a member with an MSc degree in Teaching English as a foreign language that translated the English language into Portuguese. After that, another team member with a certified (CAE and TESOL) understanding of the English language conducted the backward translation certifying the accuracy of the translation. The cultural adaptation addressed semantic, idiomatic, and conceptual concerns regarding its equivalence to the Portuguese language and cultural context. During this phase, we cross-referenced the ABALL1 educational tool with the second-grade mathematics and Portuguese language curricula. For this reason, we identified the need to add four new mathematics and seven new Portuguese language game plans, complementing the requirements of programmatic content.

Another step included the involvement of a committee of experts in clinical psychology and educational sciences to assess the acceptability of the cultural adaptation process regarding the linguistic and cultural content of each educational game (*n* = 24). A checklist to obtain qualitative feedback (e.g., *Clarity and level of understanding of the contents; Familiarity and accessibility of expressions/terms/concepts*) was developed. Based on this checklist, we have clarified several inputs regarding the contents of the activities. The main recommendations were related to (i) words and expressions more appropriate to the terminology adopted in the school curricula and (ii) expressions adjusted to the target population to ensure content accessibility. Cultural suggestions by the stakeholders were incorporated into the program and a pre-test using a sample of nine second-grade children, between 6 and 7 years old, was conducted, showing the appropriateness of the program.

### 2.2. Study design and setting

We conducted a quasi-experimental study, as sampling was not randomized, and school/class effects could not be fully controlled. A total of seven second-grade classes from the central region of Portugal, four from Gouveia and three from Vila Nova de Poiares, were selected, based on a pre-established partnership agreement, to participate in the study. A group of children who received an educational intervention program was compared to a control group without any intervention. The lack of intervention as the control only applies to the absence of play-orientated sessions provided by the “Education in Action—ABALL1” program, other usual school initiates occur concurrently. Study coordinators pre-selected classes to either IG (Intervention group, *N* = 4) or CG (Control group; *N* = 3) conditions controlling for population density and school cluster (Gouveia vs. Vila Nova de Poiares). The research design was a 2 (test group: intervention vs. control) × 2 (phase: pre- vs. post-intervention) factorial design. We examined program effects shortly after the implementation. Intervention allocation depended on school and class specificities (class size and teacher schedule) and the demographic context of each school cluster. Therefore, it was determined at the start of the study. Recruitment took place between September and November 2021. The study protocol was prepared based on the CONSORT 2010 Statement (Schulz et al., 2010).

### 2.3. Participants

**Figure 1:**
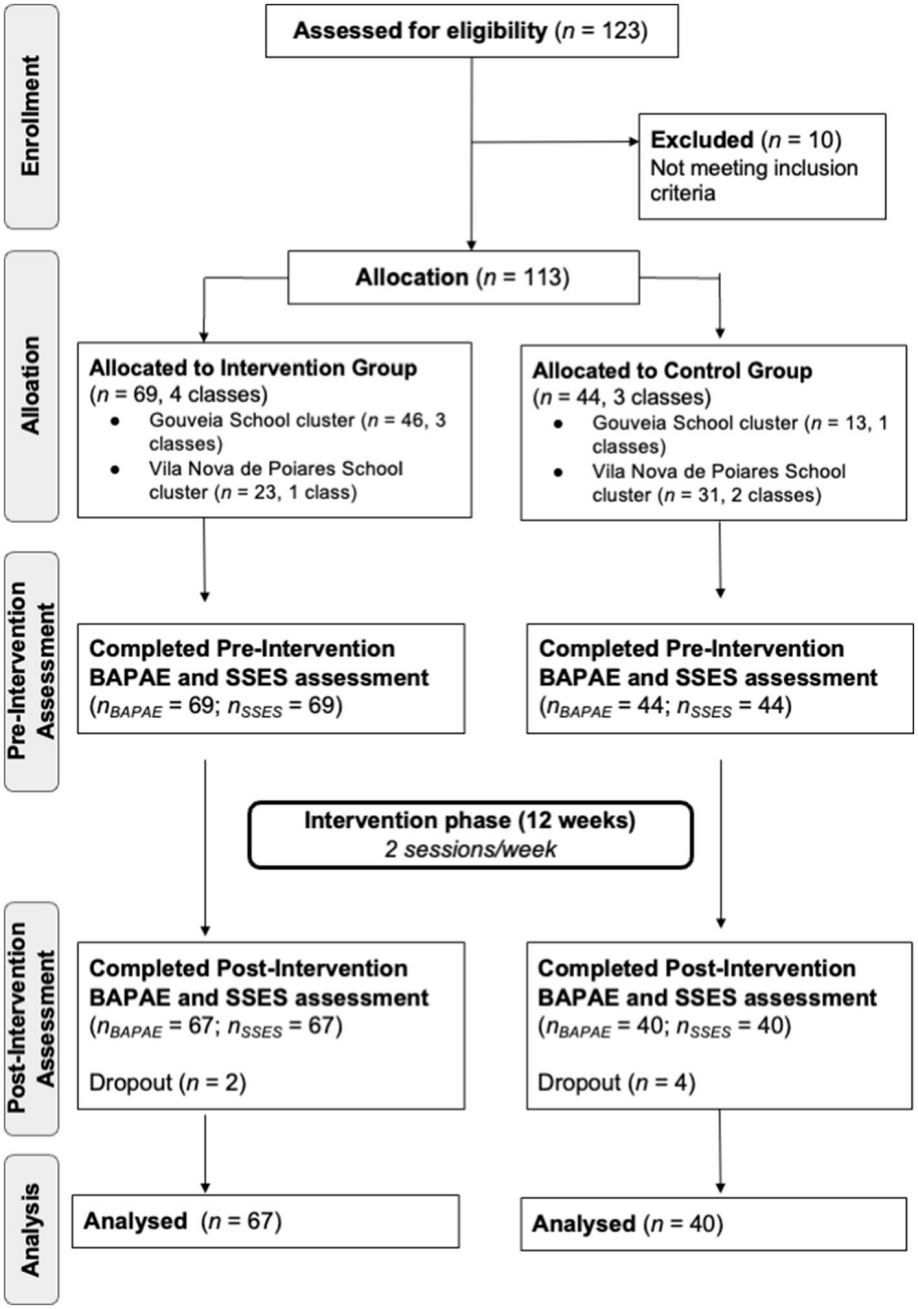
Study flow diagram.

To be included, participants had to meet the following inclusion criteria: (i) children attending second-grade level and (ii) not presenting any inability to understand and engage in the intervention program. The sample comprised a total of 113 children (59 female and 54 male) from 7 to 9 years of age (M = 7,1; SD = 0,3). As shown in the study flow diagram (Figure 1), children who attend the study inclusion criteria were allocated to IG (*n* = 69) and CG groups (*n* = 44). The groups were matched to ensure the homogeneity of the student's characteristics in the baseline. The main guardian more frequently reported was the mother. Most of them were married, employed, and had finished high school. Table 1 presents the comparison between the intervention and control groups regarding sociodemographic, educational, and socioemotional characteristics. No major differences were detected, excepting the number of siblings. Only 107 participants completed pre- and post-assessment (*n* = 6 dropouts).

**Table 1:** Participant demographics and characteristics by group (control and intervention).

**Variables**	**IG (*n = 67*) M (SD) or %**	**CG (*n = 40*) M (SD) or %**	***t* (df) or X^2^**	***P-value***
**Age**				
Child	7.1 (0.4)	7.0 (0.2)	−0.80 (105)	0.42
**BAPAE**				
Total score	70.0 (10.4)	71.0 (11.1)	0.43 (105)	0.66
Verbal comprehension	15.0 (2.3)	15.3 (2.6)	0.55 (105)	0.57
Numeracy (quantitative concepts)	13.6 (3.6)	13.5 (3.2)	−0.13 (105)	0.89
Perceptual aptitude score	41.4 (7.2)	42.2 (7.6)	0.50 (105)	0.61
**SSES**				
Cooperation	4.4 (0.7)	4.4 (0.7)	0.31 (105)	0.75
Sociability	4.6 (0.6)	4.5 (0.4)	−1.47(105)	0.14
Empathy	4.3 (0.9)	4.4 (0.7)	0.57 (105)	0.56
**Gender**				
Child (female)	55.2 %	45 %	0.04	0.82
Guardian (mothers)	86.6 %	87.5 %	< 0.001	1
**Family context**				
Number of siblings	1.3 (1.1)	0.9 (0.7)	1.98 (105)	**0.04**
Guardian marital status (married)	70.9 %	80 %	4.39	**0.03**
Guardian graduated from college	40.9 %	22.5 %	2.80	0.09
Guardian employment status (employed)	80.6 %	92.5 %	1.93	0.16

BAPAE—Battery of skills for school learning; SSES—Survey of Social and Emotional Skills; Significant P-values (< 0.05) are shown in bold and trends (< 0.10) in italics.

### 2.4. Procedure and program implementation

This study obtained ethical approval (No. 2/2019). Two school clusters participated in the project, involving seven head teachers. After the school board's approval, primary school teachers were invited to attend meetings to present the research project and for the team to understand the behavioral and social characteristics of each class and the school's internal functioning and organization. The guardians were briefed about the study, and informed consent was obtained from all participating children/students. Before the intervention, teachers completed a sociodemographic questionnaire. The evaluation protocol also included self-report measures to assess the school learning abilities and socioemotional skills, administered to children and teachers, respectively. These variables were analyzed before and after program implementation.

The “Education in Action—ABALL1” intervention program was implemented for 12 weeks, twice a week (45 to 60 min each session), from January to April 2022. The program involved a facilitator-led and was delivered in a group format with the head teacher's support of each intervention class. Two facilitators conducted the intervention, receiving previous training. The training sessions covered the use of the ABALL1 kit by one of the mentors who developed the educational tool (Glenn Andersen). Intervention fidelity was monitored by the facilitator's completion of session-specific checklists of all required activities. Monthly supervision was provided by a clinical psychologist to address intervention concerns and to review the intervention recordings providing feedback for improved implementation.

The final version of the intervention program included 24 game plans (see Table 2), namely, 12 games for each learning area (mathematics and Portuguese language).

**Table 2:** Content of the intervention program.

**Subject**	**Game**	**Thematic domain**	**Curriculum contents**	**Target socioemotional competencies**
Mathematics	Even & Odd^*^	Numbers and operations	•Natural numbers •Identification by unit number •Decimal numbering system •Addition, subtraction, and multiplication	•Problem-solving—empathy
	1001 Relay with 10	Numbers and operations	•Natural numbers •Addition, subtraction, and multiplication •Mathematical reasoning and communication	•Communication—sociability
	Dual figures	Numbers and operations	•Natural numbers •Decimal numbering system	•Communication—cooperation and sociability •Problem-solving—empathy
	Addition circle	Numbers and operations	•Natural numbers •Decimal numbering system •Addition, subtraction, and multiplication •Mathematical reasoning	•Communication—sociability •Problem-solving—empathy
	Magic triangle	Numbers and operations	•Natural numbers •Decimal numbering system •Addition and subtraction •Mathematical reasoning	•Communication—cooperation and sociability •Problem-solving—empathy
	PIN code breaker	Numbers and operations	•Natural numbers •Decimal numbering system •Addition, subtraction, and multiplication •Mathematical reasoning	•Communication—cooperation and sociability •Problem-solving—empathy
	Draws the path to …^*^	Geometry and measure	•Location and spatial orientation •Geometric figures	•Communication—cooperation and sociability •Problem-solving—empathy
	How many?	Data mining and processing	•Data display and interpretation •Point chart •Mathematical reasoning and communication	•Communication—sociability •Problem-solving—empathy
	Ratio and proportion	Numbers and operations	•Non-negative rational numbers •Mathematical reasoning and communication	•Communication—cooperation and sociability •Problem-solving—empathy
	Measurement units^*^	Geometry and measure	•Measurement: distance and length •Volume and capacity: mass •Mathematical reasoning and communication	•Communication—cooperation •Problem-solving—empathy
	Telling the time	Geometry and measure	•Time •Mathematical reasoning and communication	•Communication—cooperation •Problem-solving—empathy
	Money	Geometry and measure	•Money •Mathematical reasoning and communication	•Communication—cooperation •Problem-solving—empathy
Portuguese	Crossword	Speaking	•Discursive interaction •Spelling, vocabulary, and punctuation •Understanding and expression	•Communication—cooperation •Problem-solving—empathy
	Role-play^*^	Speaking	•Discursive interaction •Understanding and expression: role-play •Plan, produce, and evaluate their own texts	•Communication—cooperation •Problem-solving—empathy
	The pink elephant^*^	Grammar Initiation to literary education	•Listening, reading, and understanding the text •Word classes •Reading the story—second-grade National Reading Plan “The Pink Elephant”	•Communication—cooperation and sociability
	Stressed syllable^*^	Reading and writing Grammar Speaking	•Spelling and punctuation •Word classes •Understanding and expression	•Communication—cooperation and sociability
	Counting syllables^*^	Reading and writing Grammar	•Spelling and punctuation •Word classes	•Communication—cooperation and sociability •Problem-solving—empathy
	Sentence connectors^*^	Reading and writing Grammar	•Word classes •Phonemic awareness •Alphabet and graphemes	•Communication—cooperation and sociability •Problem-solving—empathy
	Word bingo	Speaking Reading and writing Grammar	•Understanding and expression •Word classes •Lexicology •Spelling and vocabulary	•Communication—cooperation and sociability •Problem-solving—empathy
	Guessing game	Speaking Reading and writing Grammar	•Spelling and punctuation •Word classes •Discourse interaction	•Communication—cooperation and sociability •Problem-solving—empathy
	Word search^*^	Initiation to literary education Grammar	•Understanding the text •Word classes •Reading literary genres belonging to the second-grade National Reading Plan	•Communication—cooperation and sociability •Problem-solving—empathy
	Picking berries	Speaking Reading and writing Grammar	•Word class •Phonemic awareness •Comprehension and expression	•Communication—cooperation and sociability
	Bouncing words	Reading and writing Grammar	•Word class •Spelling and punctuation •Understanding the text	•Communication—cooperation and sociability
	Antonyms and synonyms^*^	Speaking Grammar	•Lexicology •Discourse interaction •Comprehension and expression	•Communication—cooperation and sociability •Problem-solving—empathy

^*^These games were introduced as a complement to the requirements of the programmatic contents.

**Figure 2:**
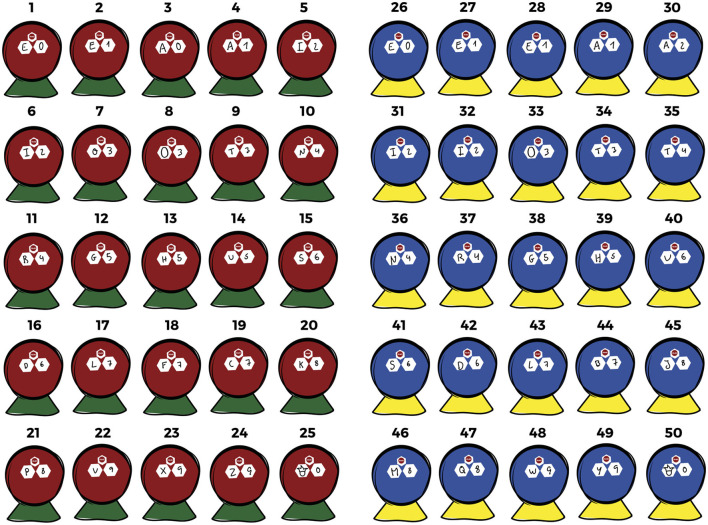
ABALL1 kit.

Support materials for program implementation included a facilitator instruction manual (Silva et al., 2022) and the ABALL1 kit (Andersen and Sandnes, 2014), composed of 50 balls (25 red and 25 blue) and 50 cones (25 green and 25 yellow) with letters (A to Z) and numbers (0 to 9) (see Figure 2).

Each session was structured in three phases: (i) an initial conversation to establish specific routines and explain the game instructions; (ii) implementation of the game enhancing the promotion of prosocial behaviors; (iii) an all-group reflection with the children, after the completion of the game, aiming to identify and summarize the educational and socioemotional skills developed. Sessions progressed from easy to difficult and were tailored to the calendarization of the programmatic content of each subject (Silva et al., 2022). In addition, after the program implementation, we conducted a focus group discussion with the teachers, who followed the intervention in the different school cohorts, and the children involved. The control group was compensated through the application of dynamics included in the program after the study finished.

### 2.5. Outcome measures

#### 2.5.1. Acceptability

Acceptability is a construct that reflects the extent to which a given intervention program is appropriate for the target population and meets their needs. Qualitative approaches are used to assess this indicator in interventions focused on promoting behavioral and social changes, including the application of focus groups (Wyatt et al., 2008; Ayala and Elder, 2011). Therefore, we have adopted this approach to encourage group discussion and obtain experiences and collective views of the teachers and children who participated in the pedagogical intervention. A semi-structured interview script was designed and applied to a subsample of second-grade children (*n* = 23) and a sample of teachers supporting the intervention program (*n* = 4). We conducted face-to-face focus group sessions with children and an online session with a teachers' group. The script included questions related to expectations, primary benefits, program suitability/associated dynamics, main facilitators and barriers to participation, perceived changes in the children's school and social-emotional skills, teachers' pedagogical practices, and future suggestions (see Table 3). The sessions were conducted by two researchers who were not involved in the implementation of the intervention program.

**Table 3:** Interview questions.

**Teacher**
1. What is your opinion about the “Education in Action—ABALL1 methodology” program as a whole? Did it correspond to your initial expectations? In what way? 2. What contents/exercises/games in the program did you find most useful for promoting skills in children? 3. Which competencies do you consider to be the most stimulated/worked with the application of the program? 4. What is your opinion about the ABALL1 kit used for the dynamics? 5. In which game(s)/exercise(s) did you feel the children had the most/least difficulty? Describe why? 6. From your perspective, what are the difficulties in implementing the program? And the facilitating aspects? 7. Would you change any aspect associated with the structure of the sessions? If yes, which ones? And why? If no, why not? 8. What changes did you identify in the children after the application of the program in terms of learning and interpersonal relationships? And in collaborative work with classmates? 9. Have you noticed any changes in your teaching practice as a result of the implementation/participation in the program? If yes, which ones? What is the greatest benefit you have derived from your participation in the program? 10. Would you recommend the application of this intervention program in other schools with students of the same age group?
**Children**
1. What have we learned from this game? 2. What did you like the most? And what did you like least? 3. Which was easier? And what was more difficult? 4. Why do you like playing ABALL1?

#### 2.5.2. Intervention adherence

The intervention adherence rate was measured by summing the total number of sessions attended by the students. Reasons for not participating in the sessions were registered by the facilitators.

#### 2.5.3. Basic learning skills

The basic learning skills were assessed using the *BAPAE*—*Battery of Skills for School Learning* (Cruz, 2019). This measure is used to assess children's skills necessary for school learning, such as verbal comprehension, numeracy, and visual-perceptive aptitude, and can be applied individually or in groups (Cruz, 2019; Reis et al., 2022). The BAPAE total score, which is composed of five subtests (verbal comprehension, quantitative concepts, spatial understanding, constancy of shape, and spatial orientation), corresponds to the cumulative sum of the number of correct answers on each subtest (the maximum score for each subtest is 20 points, except for the spatial understanding, which is 10 points, making a total of 90 points). The perceptual aptitude test is constructed by the later three subtests (spatial understanding, constancy of shape, and spatial orientation), and their cumulative sum of correct answers equate to the perceptual aptitude score (González, 2008; Cruz, 2019). The typification used in this study was conducted by applying the table of standards by schooling, which includes the 1st and 2nd years of primary education.

#### 2.5.4. Socioemotional skills

The socioemotional skills were measured through *SSES*—*Survey on Social and Emotional Skills* (OECD, 2021). This is a multi-respondent tool (self, parents, and teachers) and can be used to quantify and map the social and emotional toolkit of children and youth (8 to 15 years of age) and how they interact with their life contexts (family, school, and community) (Chernyshenko et al., 2018; Ferreira et al., 2020; OECD, 2021). Based on the intervention focus, the communication domain measures the ability to initiate and maintain social contacts and adequately convey feelings, emotions, and thoughts, with high scores indicating higher levels of communication. The problem-solving domain measures the ability to develop and explore new approaches to solve a particular problem, through trial and error, with high scores indicating higher levels of problem-solving (OECD, 2021).

Teacher report of children's social and emotional skills was assessed using a 9-item SSES survey, which asks to what extent the teacher agrees or disagrees with the description of their student on a 5-point scale (1 = “strongly disagree” to 5 = “strongly agree”). Overall mean scores are then calculated.

### 2.6. Quantitative data analysis

We used the statistical software R (R Core Team, 2022) to perform the analysis. Before any statistical analysis, normality was determined by visual assessment of the data distribution through a histogram coupled with a Shapiro test to statistically confirm the visual interpretation while heterogeneity was determined by performing a Levene test for homogeneity of variances.

The basic learning skills were analyzed using linear mixed models (lmer function from the nlme package) with a Gaussian distribution to test the effectiveness of the program. Test group (control vs. intervention), phase (before vs. after intervention), the interaction of the two, gender (female vs. male), and school cohort (Gouveia vs. Vila Nova de Poiares) were used as independent variables. We also included participant identity as a random effect to control for individual variability. Each model was validated by assessing the distribution of its residuals and Akaike information criterion (AIC) to assess how well our model fitted the data (Burnham and David, 2004; Johnson and Omland, 2004).

We calculated effect sizes (Cohen's *d*) following the procedures suggested by Payton et al. (2008). The calculation was performed as follows: first, we calculated the pre-intervention effect size (ES) for any previous intervention differences between groups and phases on each dependent variable and then subtracted this from the obtained post-intervention ES.

Regarding the socioemotional skills, cumulative link mixed models (CLMMs) were fitted with the clmm() function from the ordinal package (Christensen, 2022). The outcome of interest was cooperation, sociability, and empathy scores as ordinal, categorical variables. One factor at a time was admitted to the model in a manual stepwise forward approach. To account for the potential individual effects, participant ID entered the model as random effects. The importance of the test group (control vs. intervention), phase (before vs. after intervention), the interaction of the two, and gender (female vs. male) was added as fixed effects.

Furthermore, basic learning skills were added as covariates to infer the effect of academic skills on socioemotional scores. After each introduced variable, the AIC was assessed (Burnham and David, 2004; Johnson and Omland, 2004). To comply with model parsimony (Burnham and David, 2004), a manual stepwise backward selection procedure was carried out to address model complexity and to identify important factors. Each variable in the final, forward-selected model was due to a stepwise selection process, and the model was assessed in a manner as mentioned above.

### 2.7. Qualitative analysis

The focus group interviews were audio-recorded, fully transcribed, analyzed, and coded by two independent raters using thematic analysis, following the steps proposed by Braun and Clarke (2006), namely, (i) data familiarization, (i) formulation of initial codes, (iii) theme search, (iv) theme review, and (v) definition and naming of themes. The coding process was carried out using ATLAS.ti 22. The evaluation team reconciled discrepancies in coding through discussion. A total of 17 codes were generated and finalized. These codes were grouped into sub-themes based on similarities in the material identified. Subsequently, the sub-themes were grouped into four broader themes that reflected participants' acceptability and experience.

## 3. Results

### 3.1. Acceptability

We use focus group discussions to explore the perception of acceptability of the “Education in Action—ABALL1” program by the teachers who followed the intervention in the different school contexts and by the students involved. Overall, all participants considered the program appropriate and aligned with the content and academic requirements of the second grade. Our thematic analysis identified four themes: (i) the potential of the program in promoting school performance; (ii) the potential of the program for promoting socioemotional skills; (iii) facilitators and barriers to the program participation and implementation; and (iv) continuity and suggestions for improvement.

Regarding the program's benefits and potential for academic skills, teachers and children congruently reported that the intervention program created opportunities to acquire and consolidate knowledge in reading, writing, and arithmetic with different difficulty levels: “*I think the games served very well to apply and consolidate content, learnings that had already been given and others that had been started [...] then I went to the classroom and that week I worked on those contents instead of working on others since they had already been started in the program and I continued”* [teacher, 60 years old]; “*Because we learn better. We play easy and difficult games”* [children, 7 years old]. Teachers highlighted the program's important investment in dynamics focused on content related to synonyms, antonyms, syllables (e.g., recognition of the tonic syllable), adjectives, verbs, and sentence construction. At the level of mathematics, the positive impact of games focused on learning even and odd numbers was recognized, especially in children who had not been able to assimilate those contents in the classroom: “*I really liked the maths games [...] because it promoted and I noticed two boys who didn't make the task in the classroom, ever, and in intervention dynamic, they manifested that competence.”* [teacher, 63 years old]. The program also showed an intersection with other learning domains, which was also valued by the teachers, namely, an approach to environmental studies through games involving names of domestic and wild animals and the water cycle. Furthermore, the results suggested the role of the intervention in training cognitive skills, such as attention, concentration, memory, and reasoning, which are essential to teaching-learning processes. Through the perceptions obtained, it was also possible to verify the inclusive nature of the “Education in Action—ABALL1” program being reported that “*[...] even with children who were having more difficulties, it was much easier to teach them to play with balls, with letters, with numbers.”* [teacher, 60 years old].

Among the main benefits perceived from the intervention at the socioemotional level, there was greater cohesion in the relationships established among peers that were achieved by the teamwork provided by the activities: “*I'm very happy because my classmates helped me a lot in ABALL1.”* [children, 7 years old]. The fact that the facilitator formed different groups for the activities seemed to reinforce the sense of group. In the teacher's opinion, the interaction dynamics included in the program also potentiated a decrease in egocentrism, reinforced by the pandemic period and social isolation: “*[...] I think they also learned to work on the inter-help part, to not only want to win themselves, and also help others.”* [teacher, 60 years old]; “*I thought it was amazing that they could make the clock and tell the time when we had only talked so little [...] when one didn't know, the other helped.”* [teacher, 60 years old]. Autonomy was a positive aspect highlighted as a direct consequence of the intervention, along with the development of skills such as self-esteem, self-efficacy, and self-reflection. Importantly, the dynamic involving the final reflection on the group's performance and the encouragement of peer praise, in the opinion of all the educators, was essential for the promotion of emotional regulation and critical thinking skills: ”*They were honest, sometimes they would say: I worked very well with her, but sometimes she was a little inattentive and we would become inattentive and so on [...]. But they would start with a compliment, that less positive part was also part of the compliment, in essence.“* [teacher, 60 years old].

In addition, an analysis of perceived barriers to participation and implementation of the intervention, especially by teachers, identified contextual factors and those related to the physical conditions of the spaces as major limitations. The aftermath of the COVID-19 pandemic was noted as one of the main factors influencing initial group sharing, adherence to rules, and ability to listen to instructions: ”*[...] I think the need they have to be loud [...] is this need to talk, to talk to the other, to talk to others, to express, to tell... I think that for me the big problem was from the pandemic*.“ [teacher, 60 years old]. On the other hand, the main barriers, of a more extrinsic nature, were the high number of children in each class, which made, initially, group management difficult, and the restrictions associated with the use of the pavilion where some activities took place. The school time and the high volume of content to be covered in the curriculum were also mentioned as limitations to the implementation.

It is important to reinforce that the strategies used by the intervention facilitators, such as forming small groups with different peers, stimulating mutual help, and promoting introspection at each session, were highlighted as factors that promoted adequate management of the class, overcoming some of the initial challenges: ”*I thought it was very interesting the way she made them talk [...] she had these very funny star-style glasses [...] and the children to talk had to put the glasses on, and I thought it was very funny.“* [teacher, 60 years old]. The program, which was based on the principle ”learning through play“ and stimulating movement and physical activity during the process of knowledge acquisition (e.g., caterpillar), was also recognized as a participation benefit in Education in Action—ABALL1: “*I liked it because you could play with the balls, there were fun games and because it's cool.”* [children, 7 years old]. Some teachers reported that some dynamics continued to be implemented by students autonomously during class breaks: ”*They loved the clock, the clock was the master of games. Even now, sometimes I notice at break time, there they go doing the triangle on the floor, ready.”* [teacher, 56 years old].

Additionally, all participants in the process, teachers, and students mentioned the need for continuity of the program in the school context. The teachers reported an interest in using some of the dynamics to promote pedagogical skills, highlighting once more the transversal nature of the methodology used: “*I think it's a project that should remain in schools because we can continue with other content, other areas that we can work on [...].”* [teacher, 60 years old]. Some key suggestions for the development of future interventions involved increasing the number of sessions for a more direct approach to other subjects (e.g., citizenship). Some also mentioned the possibility of future programs being held weekly and covering children in other school years, including 1st grade: “*You should start with the first grade because they work a lot on letters and numbers and it is an easier way to learn what is not so easy, which is reading and writing. With the program, it would be a playful way to learn the process of reading and writing*.” [teacher, 60 years old].

### 3.2. Intervention adherence

Among four intervention classes, 57 students participated in the complete set of program sessions (*n* = 24). Only 10 participants completed 75% of the program sessions. The main reason for missing the intervention sessions was related to medical conditions (e.g., COVID-19, prophylactic isolation).

### 3.3. Intervention effectiveness

#### 3.3.1. Basic learning skills

**Figure 3:**
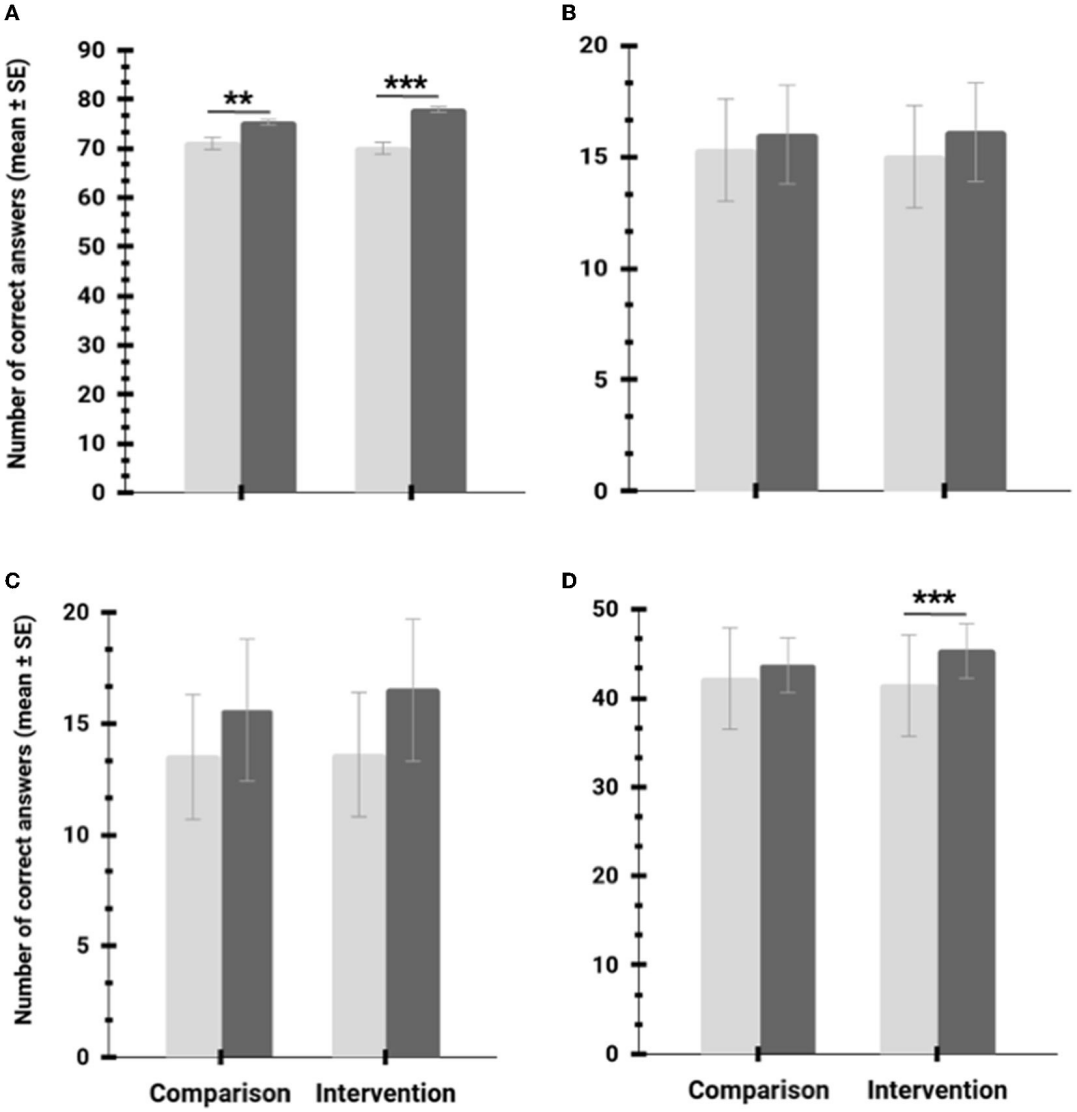
Influence of the intervention on BAPAE **(A)** total (cumulative sum of the five subtests), **(B)** verbal comprehension, **(C)** numeracy, and **(D)** perceptive aptitude scores. Light gray bars: before intervention; dark gray bars: after the intervention; ^**^P < 0.01; ^***^P < 0.001.

BAPAE total scores show a statistically significant increase after the intervention (application of the “Education in Action—ABALL1” program) independent of the test group (control vs. intervention) (see Table 4; Figure 3A). A natural increase in BAPAE total and perceptual aptitude scores was expected after the program implementation because children are continuously learning new skills. However, our analysis shows that participants from the intervention group had a significantly higher increase in overall basic learning skills than the control group (Figure 3A). Additionally, BAPAE perceptual aptitude scores of the intervention group showed a statistically significant improvement after the application of the “Education in Action—ABALL1” program (after-intervention phase). In contrast, no such significant effect could be identified by our statistical analysis in the control group (Figure 3D).

**Table 4:** Linear mixed-model analysis (LMM): BAPAE results.

**Measure**		***Estimate ±SE***	***t-value***	***P-value***	**Cohen's *d***
**BAPAE**	**Total score**				
	School cohort	1.52 ± 1.78	0.85	0.39	
	Gender	1.07 ± 1.69	0.63	0.52	
	Group	−0.30 ± 1.97	−0.15	0.87	
	Phase	4.35 ± 1.12	3.86	**< 0.001**	
	Group vs. phase	3.51 ± 1.42	2.47	**0.01**	
	*Control (Before* vs*. after)*	−4.35 ± 1.13	−3.86	**< 0.01**	0.40
	*Intervention (Before* vs*. after)*	−7.87 ± 0.87	−9.03	**< 0.001**	0.95
**Verbal comprehension**					
	School cohort	−0.49 ± 0.41	−1.19	0.23	
	Gender	−0.17 ± 0.39	−0.45	0.64	
	Group	−0.45 ± 0.49	−0.91	0.36	
	Phase	0.72 ± 0.39	1.81	*0.07*	
	Group vs. phase	0.31 ± 0.50	0.63	0.52	
**Numeracy (quantitative concepts)**					
	School cohort	0.05 ± 0.63	0.08	0.92	
	Gender	0.51 ± 0.60	0.84	0.40	
	Group	0.16 ± 0.69	0.23	0.81	
	Phase	2.10 ± 0.37	5.54	**< 0.001**	
	Group vs. phase	0.85 ± 0.47	1.78	*0.07*	
**Perceptual aptitude**					
	School cohort	1.96 ± 1.08	1.80	*0.07*	
	Gender	0.74 ± 1.03	0.72	0.47	
	Group	−0.01 ± 1.24	−0.01	0.99	
	Phase	1.52 ± 0.87	1.74	*0.08*	
	Group vs. phase	2.34 ± 1.10	2.11	**0.03**	
	*Control (before* vs. *after)*	−1.52 ± 0.87	−1.74	*0.08*	0.22
	*Intervention (before* vs. *after)*	−3.87 ± 0.67	−5.71	**< 0.001**	0.75

BAPAE—Battery of Skills for School Learning; group (control vs. intervention); phase (before vs. after intervention); significant P-values (< 0.05) are shown in bold and trends (< 0.10) in italics.

LMM of the potential effect of the school cohort (Gouveia vs. Vila Nova de Poiares), gender (female and male), group (control vs. intervention), phase (before vs. after intervention), and the interaction of the last two on the participant BAPAE scores: total (cumulative sum of the five subtests), verbal comprehension, numeracy, and perceptual aptitude.

Moreover, no significant effect of the intervention was found for verbal comprehension and numerical scores (Figures 3B, C). School cohort (Gouveia and Vila Nova de Poiares) and gender neither influenced the total score (the cumulative sum of the five subtests) nor the results obtained from the verbal comprehension, numeracy, and perceptual aptitude tests.

#### 3.3.2. Socioemotional skills

**Figure 4:**
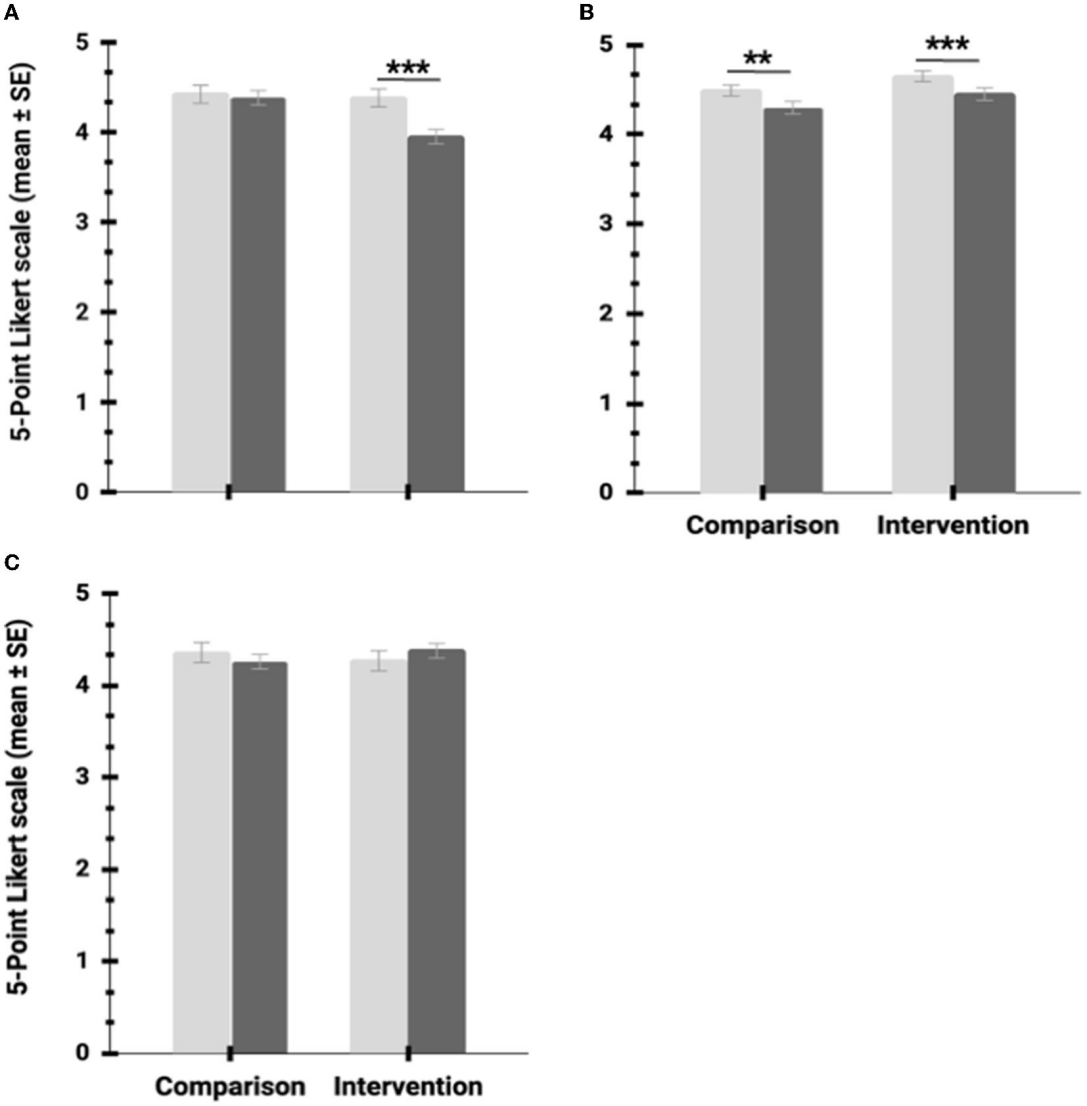
Influence of the intervention on social and emotional skills **(A)** cooperation, **(B)** sociability, and **(C)** empathy. Light gray bars: before intervention; dark gray bars: after the intervention; ^**^P < 0.01; ^***^P < 0.001.

Participants with higher BAPAE verbal comprehension had higher cooperation scores (Table 5), whereas numeracy (CLMM: z-value = 0.82; *P* = 0.40) did not show a significant effect. In addition, BAPAE perceptual aptitude scores show a trend toward a positive relationship with cooperation scores. However, cooperation scores were not influenced by phase (before vs. after intervention) and group (control vs. intervention). Nevertheless, our statistical analysis shows a significant interaction between these two dependent variables (Table 5; Figure 4A). Numeracy shows a positive relationship with sociability. However, sociability scores were not influenced by BAPAE verbal comprehension (CLMM: z-value = 1.35; *P* = 0.17) and perceptual aptitude (CLMM: z-value = 0.59; *P* = 0.55) scores. In addition, our analysis shows that phase, group, and the interaction of the two significantly affect sociability scores (Table 5). However, both control and intervention groups show decreasing sociability scores after the intervention (Figure 4B).

**Table 5:** Cumulative link mixed-model (CLMM) analysis: BAPAE scores × SSES scores.

**Measure**		***Estimate ±SE***	***z-value***	***P-value***	**Cohen's *d***
**SSES**					
**Cooperation**					
	BAPAE—perceptual aptitude score	0.07 ± 0.04	1.74	*0.08*	
	BAPAE—verbal comprehension score	0.21 ± 0.10	2.05	**0.03**	
	Gender	−2.86 ± 0.73	−3.91	**< 0.01**	
	Phase	−0.98 ± 0.57	−1.69	*0.09*	
	Group	−0.98 ± 0.77	−1.27	0.20	
	Group vs. phase	−1.45 ± 0.73	−1.98	**0.04**	
	*Control (before* vs. *after)*	0.98 ± 0.57	1.69	*0.09*	0.05
	*Intervention (before* vs. *after)*	2.43 ± 0.53	4.53	**< 0.001**	0.50
**Sociability**					
	BAPAE—numeracy score	0.34 ± 0.07	4.36	**< 0.001**	
	Gender	−0.93 ± 0.45	−2.03	**0.04**	
	Phase	−1.55 ± 0.55	−2.81	**< 0.01**	
	Group	2.31 ± 0.66	3.46	**< 0.001**	
	Group vs. phase	−2.01 ± 0.74	−2.69	**< 0.01**	
	*Control (before* vs. *after)*	1.56 ± 0.55	2.81	**< 0.01**	0.36
	*Intervention (before* vs. *after)*	3.58 ± 0.70	5.05	**< 0.001**	0.33
**Empathy**					
	BAPAE—perceptual aptitude score	0.10 ± 0.05	2.13	**0.03**	
	BAPAE—numeracy score	0.18 ± 0.09	1.93	*0.05*	
	Gender	−2.54 ± 0.74	−3.40	**< 0.001**	
	Phase	−0.61 ± 0.57	−1.06	0.28	
	Group	−0.31 ± 0.74	−0.43	0.66	
	Group vs. phase	0.20 ± 0.70	0.28	0.77	

BAPAE—Battery of Skills for School Learning; gender (female vs. male); group (control vs. intervention); phase (before vs. after intervention); significant P-values (< 0.05) are shown in bold and trends (< 0.10) in italics.

CLMM of the potential effect of BAPAE (verbal comprehension, numeracy, and perceptual aptitude) scores, gender (female and male), Group (control vs. intervention), phase (before vs. after intervention), and the interaction of the last two on the participant SSES (cooperation, sociability, and empathy) scores.

Empathy, defined as a problem-solving skill, was not influenced by how well participants understood verbal cues (BAPAE Verbal comprehension scores; CLMM: z-value = −0.65; *P* = 0.51) but by their spatial and form perception of their surroundings (BAPAE perceptual aptitude, see Table 5). Also, the ability to understand quantitative concepts (BAPAE Numeracy) shows a statistical trend toward a positive relationship with empathy scores. Our analysis did not identify a significant effect of the intervention programs on empathy scores (Figure 4C). Additionally, gender showed a significant effect on cooperation, sociability, and empathy, with female students reporting higher scores (Table 5).

## 4. Discussion

Recent studies highlight the importance of learning through play (Parker et al., 2022; Skovbjerg and Sand, 2022). Notwithstanding, the development and implementation of an intervention approach focused on promoting learning and socioemotional skills are still underexplored. This study aimed to analyze the acceptability and potential impact of an innovative educational intervention program. Our findings might suggest a positive effect of the “Education in Action—ABALL1” intervention program on second-grade children's academic skills. The student adherence rate was satisfactory, reflecting the acceptance of the program by the participants. The focus group interviews also revealed that children and teachers involved congruently appreciated and perceived the educational and socioemotional benefits of the intervention.

Our quantitative results clearly show the positive effect of the intervention in the overall basic learning skills (BAPAE) with an improvement from pre- to post-intervention assessment. Moreover, previous research has reported the benefit of using educational games allowing the transposition of theoretical knowledge, exclusive to the school context, into children's everyday life (McClelland et al., 2006; Vogt et al., 2018). Thus, our results are in line with previous research, which shows that children who participated in the program were able to apply the acquired contents to practical exercises inside and outside the classroom context, as reported by the teachers.

Specifically, the thematic qualitative analysis indicated that children developed numeracy, literacy, and problem-solving skills, although our analysis did not show statistical significance through the BAPAE subtests. These results are in line with other studies (e.g., Parker et al., 2022) that have reported the role of playful pedagogies in active engagement and proficiency in traditional learning areas, promoting holistic skills.

The learning dimension related to the perceptual attitude (BAPAE) significantly increased from pre- to post-test assessment. As expected, the improvement was higher in the intervention group compared to the control group. The specific structure and methodological approach of the “Education in Action—ABALL1” program promotes physical activity and visuospatial skills due to the peer interaction during the game and the continuous use of the material from the educational kit. Other studies, promoting physical activity, also demonstrated the role of these approaches in the stimulation of cognitive functions, such as perception, visual-spatial processing, and executive functions (Bidzan-Bluma and Lipowska, 2018).

Furthermore, our results indicate the relationship between the developed academic skills and socioemotional competencies (Greenberg et al., 2017; Mahoney et al., 2021). Our quantitative statistical analysis of the sociability scores shows that both the control and intervention groups show a statistically significant decrease in the post-intervention phase. However, the effect size (Cohen's d) of these results is small. Thus, the interpretation needs to be cautious, because the pandemic context at the time of the program implementation may have compromised the children's socioemotional skills due to the constant instability, restrictions, and occasional isolation experienced (Egan et al., 2021).

This highlights the importance of simultaneously working on learning and emotional-cognitive competencies in the school environment to promote the teaching-learning process (e.g., Denham and Brown, 2010). Hence, the “Education in Action—ABALL1” program contributed to improved learning while developing social and emotional skills, through a collaborative and inclusive perspective. These findings, supported by the children's and teachers' self-reports, highlighted the relevance of the intervention in promoting teamwork, mutual help, self-reflection, and communication.

Other pedagogical intervention programs have demonstrated similar positive effects on the skills development of school children (Raimundo et al., 2013; Appelqvist-Schmidlechner et al., 2016). However, the “Education in Action—ABALL1” program introduces a more versatile alternative by integrating essential academic skills, socioemotional skills, and the promotion of physical activity, differentiating itself from previous programs that do not favor a holistic approach. This educational program with 24 game plans also showed to be adaptable to the second-grade curricular program and learning objectives, demonstrating promising effects on the children's global competencies and strengthening a more flexible school environment. This approach offers support to the implementation of playful pedagogies in the school context, reinforcing the roles and responsibilities of the child and teacher and directly benefiting the learning process.

### 4.1. Limitations and future research

Our findings provide preliminary support for the acceptability and effectiveness of the “Education in Action—ABALL1” program, suggesting that the deliverance of this educational approach, during the school year, enhances learning and socioemotional competencies in second-grade children. However, some study limitations should be acknowledged. First, the available sample size hindered the statistical power to detect small differences between groups. This might explain the inability to detect significant quantitative changes in the competencies assessed, which were only present in the qualitative data. Second, the assessment of the intervention impact was limited, comprising only children's and head teachers' perspectives. Future studies benefit from the use of multiple reporters, such as parents and other educational actors, reducing sources of bias. Third, our study applied a non-randomized quasi-experimental design, limiting the ability to establish a causal association between intervention and main outcomes. Although randomized control trials are the “gold standard” of research (Stang, 2011), their practical implementation in the school system is complex. Moreover, two assessment points were considered during the intervention period (pre- and post-intervention), reducing the ability to determine the long-term effects of this innovative approach. Future studies should continue to invest in the implementation, adaptation, and assessment of this intervention program at other educational levels using a robust study design. Another proposal would be to adjust this intervention modality for children with disabilities ensuring learning, inclusion, and peer interaction.

## Data availability statement

The raw data supporting the conclusions of this article will be made available by the authors, without undue reservation.

## Ethics statement

The studies involving human participants were reviewed and approved by Ethical Commission of Piaget Institute No. 2/2019. Written informed consent to participate in this study was provided by the participants' legal guardian/next of kin.

## Author contributions

IS, FC-S, and SS contributed to the conception and design of the study. IS and FC-S organized the database, performed the data analysis, and wrote the first draft of the manuscript. All authors contributed to the manuscript revision, reading, and approval of the final version.
